# Competition in the German pharmacy market: an empirical analysis

**DOI:** 10.1186/1472-6963-13-407

**Published:** 2013-10-10

**Authors:** Jörg G Heinsohn, Steffen Flessa

**Affiliations:** 1 Dr Heinsohn & Partner Steuerberatungsgesellschaft, Hamburg, Germany; 2 University of Greifswald, Faculty of Law and Economics, Friedrich-Loeffler-Str. 70, D-17489, Greifswald, Germany

## Abstract

**Background:**

Pharmaceutical products are an important component of expenditure on public health insurance in the Federal Republic of Germany. For years, German policy makers have regulated public pharmacies in order to limit the increase in costs. One reform has followed another, main objective being to increase competition in the pharmacy market. It is generally assumed that an increase in competition would reduce healthcare costs. However, there is a lack of empirical proof of a stronger orientation of German public pharmacies towards competition thus far.

**Methods:**

This paper analyses the self-perceptions of owners of German public pharmacies and their orientation towards competition in the pharmacy markets. It is based on a cross-sectional survey (N = 289) and distinguishes between successful and less successful pharmacies, the location of the pharmacies (e.g. West German States and East German States) and the gender of the pharmacy owner. The data are analysed descriptively by survey items and employing bivariate and structural equation modelling.

**Results:**

The analysis reveals that the majority of owners of public pharmacies in Germany do not currently perceive very strong competitive pressure in the market. However, the innovativeness of the pharmacist is confirmed as most relevant for net revenue development and the profit margin. Some differences occur between regions, e.g. public pharmacies in West Germany have a significantly higher profit margin.

**Conclusions:**

This study provides evidence that the German healthcare reforms aimed at increasing the competition between public pharmacies in Germany have not been completely successful. Many owners of public pharmacies disregard instruments of active customer-orientated management (such as customer loyalty or an offensive position and economies of scale), which could give them a competitive advantage. However, it is clear that those pharmacists who strive for systematic and innovative management and adopt an offensive and competitive stance are quite successful. Thus, pharmacists should change their attitude and develop a more professional business model.

## Introduction

The Federal Republic of Germany has one of the most expensive health care systems in the world. The expenditure on health in 2010 amounted to EUR 287.3 billion or EUR 3,510 per capita, representing approximately 11.6% of the gross domestic product, with the vast majority (57.6%) borne by statutory health insurance [1]. A major cost factor (EUR 40.9 billion in 2010 or 14.2% of the total healthcare costs in 2010 [1]) is expenditure on pharmaceutical products. Consequently, the German Government has taken regulatory action and presented a number of healthcare reforms, aiming – at least partly – at containing the cost of pharmaceutical products. Most prominently, the “GKV-Modernisierungsgesetz” (Modernization Act for the Statutory Health Insurance System, GMG 2004) and the “Arzneimittelmarkt-Neuordnungsgesetz” (Law on the Reorganization of the Pharmaceutical Market, AMNOG 2011) were introduced to increase (inter alia) competition among public pharmacies.

German policy makers assume that competition in the pharmaceutical markets will reduce the costs of the healthcare sector. Thus, public pharmacies should compete for consumers by employing instruments of modern marketing, providing high-quality services based on modern quality management, offering tailor-made services for different target groups and implementing professional distribution policies including the most appropriate location and transport services. Thus, public pharmacists should apply instruments of active customer-orientated management (strategic management). Some authors postulate that the healthcare reforms of the last ten years have increased the orientation of public pharmacists towards competition and the application of strategic management. For instance, Stargardt, Schreyögg, and Busse [2] demonstrated that the GMG had an impact on the retail prices of five selected over-the-counter (OTC) products. However, there is no evidence that this impact is a matter of fact for all pharmacies and their complete spectrum of products [3-6].

This paper analyses the perceived economic situation as well as the competitive behaviour of public pharmacies in the outpatient sector in Germany. It examines the effects of the competitive situation, innovativeness, location and willingness to co-operate on commercial success and draws conclusions in relation to the need and readiness to use strategic methods of business management in pharmacies. For this purpose, the next section provides some basic information on the German healthcare system. Subsequently, the methodology is presented. This is followed by the results and discussion.

## Background

The following analysis refers exclusively to the pharmaceutical expenditure of the statutory health insurance sector in Germany based on the “Sozialgesetzbuch V” (Social Act V, SGB V), i.e. the special rules for the proportion of the German population within private insurance – approximately 15% (about 12 million out of the total population of 82.3 million) – are not reflected. Statutory health insurance in Germany is based on several principles relevant to this analysis. Most importantly, in every budget period the income of the insurance must recover the costs. Thus, increasing costs will result in the raising of the contribution rate, which is not seen as favourable for the economy.

In addition, the individual risk of disease (e.g. previous illnesses, age) has no influence on the contribution rate. However, health insurance expenditure does not comprise the total expenditure on health. For example, private households have to make a co-payment for drugs themselves and a significant proportion of the expenditure on medicines is not recoverable. Most patients obtain their pharmaceutical products from a public pharmacy. A minor share is provided by institutional pharmacies (e.g. a hospital pharmacy after admission) or is purchased from normal retail shops. Drugs are frequently prescribed by physicians and these can only be bought from public pharmacies. In principle, the health insurance pays for the pharmaceutical products, but there are a number of exemptions.

First, prescribed drugs are subject to a surcharge amounting to 10% of the pharmacy sales price with a minimum of EUR 5 and a maximum of EUR 10 per package. Secondly, non-prescription OTC drugs which are sold in pharmacies have essentially been excluded from reimbursement since 2004 (with the exception of a list of non-prescription drugs that are reimbursed). Drugs sold at normal retail shops are non-prescription and are not paid for by the insurance.

Expenditure on healthcare services has grown steadily and there is a strong literature explaining the causes, such as medical-technical progress and demographic developments [7,8]. In particular, the influence of age, both on morbidity and the consumption of pharmaceutical products, is of considerable relevance in the increase in healthcare costs and the consumption of pharmaceuticals [9]. The group of insured persons above the age of 60 constitutes 26.8% (all figures from 2008) of the insured population, but accounts for 54% of the expenditure on pharmaceutical products covered by statutory health insurance [10]. In contrast, the contributions of this group of insured persons cover only 22.1% of the income of statutory health insurance [11].

From 1991 to 2009, the share of the population above 65 years increased from 14.9% to 20.7% [1]. By 2060, the population in the 65+ age group is expected to comprise a share of 34% [12], so that we can expect increasing expenditure on pharmaceutical products. Consequently, health policy makers frequently call for increased efficiency in pharmaceutical markets and design healthcare reforms accordingly.

The fundamental assumption of all healthcare reforms in Germany is that public pharmacies competing in rather free markets will serve their customers better and at a more favourable price than in a market where the legislator prohibits competition. Traditionally, the prices of pharmaceutical products were fixed in Germany. Pharmacists could only compete with others by offering good consultation services to their clients and they could not transfer their innovative concepts to other pharmacies because each pharmacist was allowed to operate only one pharmacy (prohibition of multiple ownerships). Mail-order business was also prohibited.

Since 2004, the legislator has abolished these restrictions to enhance competition. For instance, pharmacy owners have been allowed to operate up to three further public pharmacies (so-called branch pharmacies) in addition to their main pharmacy. At the same time, mail-order purchases have become possible. The only major restriction that has not yet been abolished is the prohibition of third-party ownership, i.e. only professional pharmacists with a university degree in pharmaceutics and an additional internship are allowed to own and run public pharmacies. This condition was recently endorsed by the European Court of Justice.

The remuneration of public pharmacies is based on a so-called combi-model. Pharmacists receive a fixed pharmacy fee and a linear mark-up of 3% for pharmaceutical products per package. From 2004, the fixed pharmacy fee was EUR 8.10, but it was recently cut back by a pharmacy discount of EUR 2.30. Since 2004, the prices of OTC products have been based on a regressive mark-up scheme. Therefore, price competition is only feasible outside the assortment of prescribed pharmaceutical products. In principle, prescribed drugs cost the same for healthcare insurance and for the patient in every pharmacy in Germany. Thus, competition must focus on services, information, location and customer orientation. However, we do not know whether the Government’s intention to increase competition is actually perceived by the pharmacists and whether these reforms are having any impact on their management practices. This study was designed to provide some insights into the impact of the healthcare reforms on the management of public pharmacies.

## Methods

The analysis in this study is based on a questionnaire asking for facts, opinions and perceptions of pharmacists running public pharmacies in the Federal Republic of Germany. Mail-order pharmacies were not included. If a pharmacist runs more than one outlet, each outlet is counted as a separate public pharmacy.

The questionnaire included closed and open questions. After a pre-test, 10% of German public pharmacies (i.e. 2,168 out of 21,679 pharmacies) were randomly selected and contacted in writing. To ensure a sufficient response rate, all seventeen Regional Chambers of Pharmacists were informed of the study and asked for their support. The survey was undertaken from June to October 2008. Four to six weeks after the respective dispatch date, a reminder was sent to the pharmacists. In cases where e-mail addresses were available, the reminder was sent electronically for reasons of cost; both the questionnaire and the voucher were provided as PDF files. In the case of reminders by letter, the pharmacists were given the possibility of downloading the questionnaire. Due to the anonymity afforded to the respondents, reminders had to be sent to all the pharmacists.

The questionnaire comprised 37 questions (see Additional file 1) including:

•Duration of existence of pharmacy

•Establishment/take-over mode

•Multiple ownership (if any)

•Localization of pharmacy

•Size of catchment population

•Local purchasing power

•Planned or actual change of location

•Competitive position (perceived number of competitors, perceived competitive pressure, evaluation of own competitive position)

•Attitude towards competitors (offensive attitude)

•Willingness or plans for cooperation

•Active customer-orientated management

•Success factors (net revenue development, profit margin)

•Frequency of recommendation of pharmaceutical products for self-medication and information on best-selling self-medication areas

•Sales and turnover of different segments (economies of scale)

•Personal assessment of regulations in the pharmacy market

The central theoretical basis of the confirmatory study is the search for success factors [13-15]. Furthermore, our research is based on concepts of indirect performance measurement, primarily competitive forces and the industry structure analysis of Porter [16,17] with its starting points of the market-based view and the resource-based view. The central methodological positioning is based on Popper’s propensity model [18].

The analysis was performed using uni-, bi- and multivariate models for the different items, as well as structural equation modelling (SEM). The main statistics used were Spearman’s rank correlation (R), the chi-square test and the Mann–Whitney U test. These procedures are standard in economic and social research and can be used even in the case of inhomogeneous data dispersions and when the level of measurement is relatively low (i.e. ordinal data). The statistical analysis presents the perceived competitive pressure, a number of selected hypothesis tests between “Economic Success” and geographical pattern, business performance, active customer-orientated management, and finally cooperation strategy.

In addition, we developed a SEM [19] in order to confirm the importance of each factor influencing the economic success of public pharmacies. The focus of the statistical analysis is the construct “Active Customer-Orientated Management”, represented by the indicators “General Intention to Develop”, “Customer Loyalty”, “Development of Customer Orientation/Customer Loyalty”, “More Innovation” and “Offensive Attitude”, the opposite to defensive attitude [20] . We also analyses the relation between these indicators and economic success. The H_**0**_-hypothesis between the construct economic success and the indicators was in all cases that there is no causality. We estimated the SEM using nonlinear universal relational modelling (NEUSREL) [21,22] as we were primarily working with ordinal data and therefore on the level of measurement defined above. The ordinal data did not allow the application of standard procedures but called for more advanced statistical procedures with more complex models in a structural-analytical way [23]. This is one reason for preferring NEUSREL as unlike the most popular statistical packages, LISREL and AMOS, a mix of scales (nominal, ordinal or metric) is allowed and Gaussian distribution is not assumed. NEUSREL is based on the statistical package PLS with additional features and Bayesian neural networks [21,24,25]. For example, NEUSREL is able to identify unknown paths, model interaction between monitored or manifest and unmonitored or latent variables and identify unknown nonlinearities [21,22].

Another essential component is the SEM process. This process focused on two steps, the estimation of the measurement model and the fitting of the SEM. In our pragmatic model development approach, we combined confirmatory and exploratory purposes. There is no prior empirical study with conceptualized latent variables. For these – exploratory – purposes a soft modelling package with soft or relaxed assumptions about data is used. An alternative modelling approach was not indicated by the current state of research. However an accepted model is only a confirmed or not-disconfirmed model.

A further important reason for choosing a variance-based modelling and variance-based statistical package respectively is the requirements of a small representative sample. The literature gives no uniform measurement for a sufficient sample size [15]. In the case of multivariate and structural-analytical aims, the recommendations in the literature vary considerably, from at least 100 (or preferably 200) to five cases per parameter to be estimated, or a relation of 2:1 between parameters to be estimated and case number [26,27]. In the context of PLS, Chin indicates a heuristic with the relation 10:1 between parameters to be estimated and case number, or the decuple of the largest quantity of indicators [28]. A sample of less than 100 is indicated as appropriate in the literature in the context of PLS [29], but with the proviso that “PLS is not a silver bullet to be used with samples of any size!” [30]. The conditions stated regarding sample size were fulfilled in our empirical analysis, which drew on data from 289 German pharmacies (see the Results section below).

**Figure 1:**
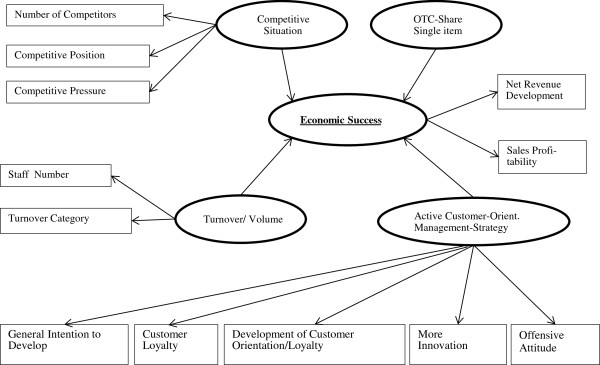
Structural Equation Model (SEM).

As Figure 1 shows, we assume that the economic success of public pharmacies is determined to a considerable extent by the competitive environment being a composite of the number of competitors, competitive position and competitive pressure. Furthermore, we assume that large pharmacies and pharmacies with a high purchasing capacity realize an economically stronger development. We also suppose that public pharmacies with more innovation, an offensive attitude towards competitors and a general customer-orientated strategy (and with a focus on OTC products, measured as a share of total turnover) will be more successful.

In the results section, we use Cronbach’s alpha, composite reliability and average variance extracted (AVE) to describe the fit of the model. Cronbach’s alpha coefficient is one of the most widely used estimators for the reliability of tests and a measure of internal consistency or reliability [31,32]. If the test is not unidimensional, it is likely that the true reliability will be underestimated [33]. In exploratory cases, a value equal to or higher than 0.6 for the Cronbach’s alpha coefficient is considered satisfactory [34,35]. As Cronbach’s alpha coefficient is only allowed under the assumption of linearity and equal loadings for all manifest variables [26,32,36], composite reliability can be considered a better criterion for reliability [28]. Composite reliability above 0.7 indicates high reliability [33,36]. However, Bagozzi [37] and other authors [38] have accepted a minimum level of 0.6.

Cronbach’s alpha coefficient and composite reliability are instruments used to measure the compliance of reflective indicators. The third model parameter, the AVE, measures the amount of variance that is captured by the construct. The AVE should be a minimum level of more than 0.5 [37-39].

Finally we use a less common parameter, Scott’s homogeneity ratio, which measures the average inter-item correlation. If Scott’s homogeneity ratio is approximately zero, then the manifest variables do not distinguish the constructs (or latent variables). If Scott’s homogeneity ratio is less than 0.4 or between 0.2 and 0.4, it is considered acceptable [40,41].

## Results

The results are presented in several sub-sections. We start with a description of the sample. We then provide some insights into the perceived competitive pressure in relation to public pharmacies in Germany. This is followed by a number of bivariate analyses (geographical pattern, business orientation) and the results derived from the SEM.

### Description of the sample

Of the 21,679 stationary public pharmacies (at the time of the study, excluding mail-order pharmacies and including branches), 10% were randomly selected. In total, 291 pharmacies responded and 289 (13.33%) questionnaires could be used. Therefore, 1.33% of the established pharmacies in Germany contributed to the insights provided in this study. We consider this a rather strong sample size in comparison to the above-mentioned requirements.

The correlation between our study sample and the population in each state is highly significant (Spearman rank R = 0.87, p < 0.001). Thus, the sample can be considered geographically representative. Table 1 shows some baseline characteristics of the sample of public pharmacies in Germany.

**Table 1:** Baseline characteristics of the sample

		**%**
**Number of inhabitants at the location**	**< 5,000**	**18.20**
	**5,000 – 10,000**	**20.60**
	**10,000 – 50,000**	**32.10**
	**50,000 – 100,000**	**8.40**
	**100,000 – 500,000**	**13.60**
	**> 500,000**	**6.30**
	**Miscellaneous cases**	**0.80**
		
**Classification of sales volumes [T€]**	**< 1,250**	**31.60**
	**1,250 – 2,400**	**52.20**
	**> 2,400**	**16.20**
		**%**
**Owner**	**Female**	**43.30**
	**Male**	**56.70**

Unfortunately, no official statistics on the gender of pharmacists running public pharmacies exist. Consequently, it is difficult to assess whether our sample is representative. The association of German pharmacists, ABDA, provides data on the gender ratio of this profession, irrespective of whether they are employed or run their own pharmacies [42]. According to these statistics, 33.4% of German pharmacists are male, 66.6% are female (year 2007). Our own sample shows that 56.7% of pharmacists running a public pharmacy are male, 43.3% female. However, these figures cannot be compared as it is likely that a higher proportion of male pharmacists run their own public pharmacies than female pharmacists.

### Competition

A number of questions aimed to determine the perceived level of competition in the public pharmacy market in Germany. As Table 2 shows, the number of direct competitors in the catchment area of public pharmacies is still limited. The majority of pharmacies have three or fewer competitors, although a few have more than ten. Consequently, most public pharmacies view their own position in the market as acceptable or good and perceive the pressure from their main competitor as moderate or even low. There is no single year in which the competitive pressure increased dramatically, but the GMG (2004) seems to have had the greatest impact on the competitive situation. The responses of the public pharmacists on the (limited) increase in competitive pressure are quite conservative. They try to gain a competitive edge by providing high-quality consultancy services to their clients and offering some after-sales services, such as transport.

**Table 2:** Perceived competition on German public pharmacy market (rounded)

**Question**	**Mode**
How many public pharmacies are you directly competing with?	0: 13.5%
	1: 18%
	2–3: 23%
	4–6: 18%
	7–10: 9%
	>10: 20%
How do you assess your competitiveness on the market?	Very good: 5%
	Good: 33%
	Acceptable: 31%
	Difficult: 26%
	Very difficult: 6%
How do you assess the competitive pressure of your main competitor?	Very low: 4%
	Low: 16%
	Moderate: 51%
	High: 19%
	Very high: 10%
Since when do you perceive an increase competitive pressure?	Before 2003: 28%
	2003: 5%
	2004: 26%
	2005: 14%
	2006: 12%
	2007: 10%
	2008: 6%
In which field are you better than your competitors?	Innovation: 38%
	Quality of products: 21%
	Advise: 74%
	After sales service: 57%
	Price: 21%

Consequently, we can state that the level of competition in the public pharmacy market is not as high as one would expect. Even after a series of laws aimed at increasing the competition, the majority of pharmacists are still in a situation of “smooth” water.

### Geographical pattern

Based on this sample, we undertook a number of bivariate analyses in order to determine the correlation between parameters. In a first step, we determined the impact of the location of a public pharmacy on business performance. For instance, we wanted to know whether there is any significant difference in terms of economic success between public pharmacies in East Germany (the former German Democratic Republic) and West Germany. The hypothesis that there would be no difference was tested by comparing the location (West vs. East) with the net revenue development and profit margin of public pharmacies. Based on a non-parametric Mann–Whitney U test, we can show that there is no systematic pattern in the effect on net revenue development (transformed Z-value from the U test, absolute = 0.15, p = 0.44 one-way; two-sided testing results in the same conclusion, p = 0.88). However, there is a significant effect on the profit margin, namely that for public pharmacies in West Germany (N = 218) a higher profit margin was quoted than for public pharmacies in Eastern Germany (N = 52) (transformed Z-value from the U test, absolute = 2.0; p < 0.05 one-way; two-sided testing results in the same conclusion, p < 0.05).

In addition, we wanted to examine whether there are any geographical differences concerning forms of cooperation. For this element of the analysis, we distinguish between North (Schleswig-Holstein, Brandenburg–Berlin, Hamburg, Saxony-Anhalt, Bremen, Lower Saxony, Mecklenburg–Western Pomerania, and North Rhine-Westphalia) and South (Bavaria, Baden-Württemberg, Saarland, Rhineland-Palatinate, Hessen, Thuringia, and Saxony), as well as between East and West Germany. According to the Mann–Whitney U and the chi-square tests, there was no geographic pattern in terms of cooperation as actually practiced by the pharmacists, either for East–West (chi-square = 6.4, df = 4, p = 0.17), or for North–South (chi-square = 6.4, df = 4, p = 0.17), or for North–South without the Eastern States (chi-square = 1.5, df = 4, p = 0.82). The relevant statistical tests merely confirm a uniform level of cooperation 22 years after German reunification in all parts of the country.

In addition, we expected that the turnover of public pharmacies in rural places would be much lower than in cities [43,44]. However, the data do not support this hypothesis. The variables “Population Size” and “Turnover” were not significantly correlated (chi-square = 1.9, df = 2 at N = 226, p = 0.38). Also, the status of “Major City” showed no link with the turnover of the pharmacies surveyed. A further inspection of the data revealed that the highest turnover tends to be achieved in smaller and medium-sized locations, but this is not significant. This result corresponds to the findings of Schöffski [45]. However, Schöffski’s study focused on the implementation of regulation and deregulation measures for pharmaceutical products and included only descriptive statistical analysis.

### Business performance

Based on economic theory, we would expect a correlation between the competitive environment and the business performance of a public pharmacy, i.e. strong competition should have a negative influence on turnover and the profit margin. Based on our survey, the results are not definitive. First, we compared the number of relevant competitors (stated by pharmacists) with net revenue development and the profit margin (“Economic Success”). However, it seems that none of these outcome parameters is significantly correlated with the number of competitors. However, when we asked the pharmacists about their perceptions of competitive pressure, the results changed. The perceived competitive environment in the pharmacy market was significantly negatively correlated with business performance (R = -0.38, p < 0.001; R = -0.26, p < 0.001). The perceived competitive pressure from main competitors showed the same result (R = -0.30, p < 0.001; R = -0.20, p < 0.001). It therefore seems that the number of competitors is no indicator of competitive pressure and that for many pharmacists competition is still not an issue at all.

In addition, we would expect that the purchasing power of the catchment population would have a strong impact on the business performance of public pharmacies, in particular in the self-medication segment [45,46]. First, we analysed the relationship between purchasing power and the share of OTC and prescribed drugs in total turnover. As expected, there is a positive and statistically significant correlation between purchasing power and OTC turnover as a share of total turnover (R = 0.21, p < 0.01). At the same time, the relationship between purchasing power and the turnover of prescribed medicines as a share of total turnover became clearly negative (R = -0.33, p < 0.001). In other words, public pharmacies rely strongly on prescribed drugs in catchment areas with poor purchasing power, such as in Eastern Germany.

Second, we analysed whether the perceived importance of the OTC segment (asked by the question “How often do you recommend non-prescribed pharmaceuticals for self-medication when advising customers?”) has an influence on the profit margin. We can confirm that the orientation towards OTC has an impact on the profit margins of public pharmacies (R = 0.22, p < 0.001).

Third, we directly related the purchasing power of the catchment areas to business performance. As expected, net revenue development correlated positively with purchasing power (R = 0.14), but it was not significant. The relationship between profit margin and purchasing power was even weaker (R = 0.04). Thus, competition for OTC clients with strong purchasing power is of relevance for the public pharmacies, but generally the business performance of German public pharmacies does not depend strongly on the purchasing power of its catchment population.

Another assumption of business administration is that a bigger business unit will have a higher profit margin: the fixed costs of a public pharmacy are high so increased sales will reduce the cost per unit and increase the profit per unit. We used turnover and number of employees as proxies for the size of the pharmacy. Our results support this assumption as the turnover of the pharmacy and the profit margin are highly and significantly correlated (R = 0.23, p < 0.001). The same results hold true if the number of employees is taken as a proxy of size (R = 0.68, p < 0.001).

### Active customer-orientated management

One would expect that pharmacists demonstrating greater innovativeness, good levels of customer consultation and other services and a higher marketing orientation would have stronger revenue increases and a higher profit margin. Consequently, we asked the pharmacists for their self-assessment of their innovativeness (“More Innovation”), the quality of their customer services and their marketing orientation in comparison with their competitors. As Table 3 shows, revenue development correlates to self-perceived innovativeness, customer consultation, other customer services, marketing orientation, offensive attitude, general intention to develop and development of customer orientation/loyalty. Only the indicator “More Innovation” is correlated with both success criteria. However, the correlation is lower and by far less likely to be significant for the profit margin than for revenue development.

**Table 3:** Bivariate analysis (selected parameters and business performance)

	**Net revenue development**	**Profit margin**
More innovation	R = 0.37	R = 0.28
	N = 274	N = 266
	p < 0.001	p < 0.05
Customer consulting	R = 0.2	R = 0.11
	N = 273	N = 265
	p < 0.001	p < 0.05
Customer service	R = 0.23	R = 0.18
	N = 274	N = 266
	p < 0.001	p < 0.01
Marketing orientation	R = 0.3	R = 0.09
	N = 273	N = 265
	p < 0.0012	p < 0.07
Offensive attitude	R = 0.21	R = 0.05
	N = 279	N = 271
	p < 0.001	p < 0.19
General intention to develop	R = 0.25	R = 0.18
	N = 280	N = 271
	p < 0.001	p < 0.01
Development of customer orientation/loyalty	R = 0.21	R = 0.11
	N = 282	N = 273
	p < 0.001	p < 0.05

Customer consultation, customer service and marketing orientation represent the indicator “Customer Loyalty” in the SEM. The dimension “Development of Customer Orientation/Loyalty” covers the self-perceived intention to extend customer satisfaction (e.g. through personnel training) and the organization of events for customers/patients concerning health-related topics.

### Cooperation strategy

A final strategic option is cooperation with other public pharmacies. In “normal” markets, one would expect that cooperating business units would have higher turnover and profit margins. However, our analysis detected no systematic relationships between cooperation intensity and profit margins (R = 0.09, N = 276, p = 0.08). Likewise, there are no homogeneous or drastic correlations between the degree of cooperation and the various considerations of turnover share surveyed. In detail, the results are as follows, broken down by turnover share:

•Turnover, supplementary range (body care products/food supplements): R = -0.09, N = 208, p = 0.11, one-way.

•Prescription turnover (prescription pharmaceuticals): R = 0.03, N = 210, p = 0.32, one-way.

•Prescription turnover (OTC pharmaceuticals): R = -0.12, N = 192, p < 0.05, one-way.

•Final consumer sales (OTC pharmaceuticals): R = 0.10, N = 198, p = 0.08, one-way.

### Structural equation model (SEM)

**Figure 2:**
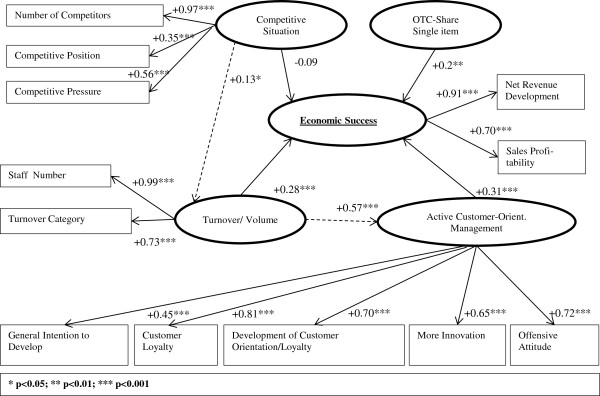
Results of the Structural Equation Model (SEM).

As described in the methodology, the SEM was used to determine the importance of the different factors influencing the business success of public pharmacies. The model, as given in Figure 2, is acceptable with a GoF (Goodness of Fit) of R^2^ = 42% with ten iterations [21]. The most important model parameters are presented in Table 4.

**Table 4:** Selected quality criteria of the Structural Equation Model (SEM)

	**Cronbachs-alpha**	**Composite reliability (CR)**	**Average explained variance (AEV)**	**Scott’s homogeneity**
Economic success	0.63	0.89	0.71	0.71
Competitive situation	0.61	0.60	0.47	0.34
Turnover/volume	0.73	0.85	0.67	0.59
OTC focus	-	-	-	-
Active customer-orientated management	0.71	0.55	0.43	0.44

The SEM shows no clear-cut distinction between the different criteria. The minimum level for Cronbach’s alpha coefficient in exploratory cases, according to Bagozzi [35,37] and Nunnally [34], and Scott’s homogeneity ratio [40,41] are completely fulfilled.

For both criteria, the composite reliability and the AVE, the construct “Active Customer-Orientated Management” is critical. It should be noted that the value for the composite reliability for the construct “Active Customer-Orientated Management” at 0.55 is borderline. Therefore, it must be acknowledged that this result does not meet the ideal condition (here GFO/R^2^ > 50%; Cronbach’s alpha coefficient ≥ 0.6; composite reliability ≥ 0.6; AVE ≥ 0.5 and Scott’s homogeneity ratio ≥ .2). As this is the first SEM exercise in the pharmacy field in Germany, the results are relative. It is the starting point for further analysis and the values of the different quality criteria presented can be judged adequate for the purposes of an exploratory study.

The SEM, calculated with NEUSREL [21,22] using 11 variables and standardized correlations, is shown in Figure 2. The model expresses a set of important partial hypotheses concerning direct and indirect relations between monitored or manifest and unmonitored or latent variables. The rectangles represent manifest variables – for example sales profitability – and the ovals represent the latent variables, which are abstract reflective constructs, with the different variables or indicators [20]. Solid arrows indicate hypothesized direct effects and broken arrows indicate indirect significant effects. In the case of OTC focus, a single item with a factor status is involved. In addition, Figure 2 depicts the correlation between the dimensions and the respective latent constructs. The significantly negative correlation between perceived competitive pressure and business performance (R = -0.38, p < 0.001; R = -0.26, p < 0.001) based on the univariate-level analysis changed in the SEM to a small non-significant direct effect. Otherwise the model identifies two additional indirect effects, especially that between turnover/volume and active customer-orientated management. In contrast to this effect, the competitive environment and the turnover/volume indicate no substantial relationship. This may be a reflection of the regulations within the German pharmacy market outlined in the introduction to this paper, i.e. the neutralization of competitive action.

Finally, Table 5 shows that some indirect effects between the latent constructs were not significant.

**Table 5:** Non-significant effects

**Construct/variables**		**Effects**
Competitive situation	OTC	0.06
Competitive situation	Strategic management	0.08
Turnover/volume	OTC	0.08
OTC	Strategic management	0.04

## Discussion

The public pharmacy market in Germany is undergoing a process of deregulation. This is predicted to increase competitive pressure and raise the competitive orientation of pharmacies. The literature states that there should be growing competition and that this should have an impact on the business orientation of pharmacists [3-6,45], but there is no proof of this. This paper has presented the first empirical cross-sectional survey (N = 289) of the competitive orientation of public pharmacies in Germany since the start of deregulation.

The analysis shows that there is no North–South or East–West dichotomy to any great extent. Thus, we cannot detect aspects of competitiveness that determine economic success in relation to particular locations. However, for the success factor “Profit Margin” a significant effect for geographic location was registered: higher profit margins were declared by West German pharmacies (N = 218) than by East German pharmacies (N = 52). Thus, the null hypothesis of a lack of difference is rejected in part because economic success operationalized in relation to the pharmacy profit margin was higher in the “old” federal states than in the “new” federal states.

In many cases, the tests of the hypotheses showed a disparity between the success factors “Net Revenue Development” and “Sales Profitability” and the strength of the factors under investigation. In relation to this, the realities of the regulations within the German pharmacy market are briefly outlined in the introduction and become especially evident when examining purchasing power and the opposite effects in the segment of prescription pharmaceutical products (Rx) and the self-medication segment (OTC) of the pharmacies. To some extent, these opposing effects reflect the principle of solidarity and the resulting moral hazard [26,47]. On the one hand, description of purchasing power in this study emphasizes the declining development of the self-medication market. This indication runs contrary to opinions expressed in the literature in the context of the postulated increase in patient autonomy and the growing self-medication market supposed to result from this [3,44,45]. On the other hand, the results for purchasing power and the often postulated patient autonomy at least allow the hypothesis that the trend towards a growing patient autonomy is linked to household budgets.

The relation between profit margin and the OTC segment is quite strong (R = 0.22, p < 0.001). It remains to be seen what influence AMNOG will have on profit margins. With AMNOG, a new distribution remuneration system for the wholesale trade was introduced as of 1 January 2012, comparable to the systematization of distribution remuneration for pharmacies in the so-called combi-model. The combi-model for the wholesale trade consists of a percentage surcharge of 3.15% and a fixed remuneration of EUR 0.70 per package with an upper limit of EUR 37.80. Currently, pharmaceutical wholesalers are using these changes to make considerable reductions in discounts, although the revision leads to a loss in margin for wholesale pharmaceuticals only from a manufacturer price of upwards of EUR 18.19. Due to the turnover structure of many pharmacies with a major drug share in the segment up to EUR 18.19, the reduction in discounts is not understandable. The influence of the revision in the distribution remuneration of pharmaceutical wholesalers on the OTC proportion in pharmacies in a statistical sense remains to be seen.

The missing correlation between membership in a pharmacy cooperation and a higher profit margin contradicts traditional economic theory. A possible explanation can be deduced from the behavioural assumptions and the efficiency criterion of transaction cost theory, as well as the conflict between “reduction in uncertainty” and “loss of autonomy” from the perspective of resource dependency. The efficiency criterion assumes the traceability or transparency of the benefits derived from pharmacy cooperation. In the simplest case of a purchasing alliance, the apparent lack of transparency of the purchasing advantages could be an indication of pharmacists’ perceptions of cooperation. If a purchasing advantage is in principle easy to measure, in the regulatory context of the pharmacy market it is nonetheless difficult to indicate the economic significance to members. Transparency, in terms of perception, gains an additional dimension in this connection. The behavioural dimension of opportunistic behaviour in transaction cost theory and the loss of autonomy from the perspective of resource dependence offer – through a common intersection – another potential explanatory approach. Potentially, the interests of the individual members of a pharmacy cooperation do not have priority, but rather those of the cooperation do.

The SEM shows that there are reflective indicators, such as self-perceived innovativeness, the development of customer orientation/loyalty, customer loyalty and offensive attitude, which are correlated with the construct “Active Customer-Orientated Management”. Furthermore, the SEM, with the respective effect sizes of the manifest variables on the latent construct “Economic Success” illustrate especially the relationship between “Competitive Situation”, “Turnover/Volume” and “Active Customer-Orientated Management”.

However, our study faces a number of shortcomings. First, the sample size of 289 is only 1.33% of the total number of public pharmacies in Germany. In part we compensated for this shortcoming by using the soft modelling statistical package, NEUSREL. However, there is no other recent study with such a high number of questionnaires available for evaluation or that has constructed an SEM to assess “Economic Success” in the German public pharmacy market.

Another shortcoming is that we focused primarily on the self-perceptions of owners of public pharmacies. One might assume that objective facts would lead to results that are more reliable than subjective self-perceptions. However, it is clear that the perceptions of pharmacists drive decisions, not objective facts, particularly if the decision makers are not fully aware of these aspects. Thus, stressing self-perceptions is indeed the most appropriate way to determine the driving factors. The comments from the pre-test were incorporated into the questionnaire and thus all items were formulated clearly and easy to understand. Furthermore, particular attention was paid to ensuring that theoretical and content-relevant aspects were duly recorded. The reply density of all items, including open questions, was thoroughly reviewed by the pharmacists. There was no reference to a lack of comprehensibility in the questionnaire or similar signals for artefacts, such as distortions in the phenomenon “Social Desirability”. The response bias or non-response bias in the framework of a survey with specialists can be disregarded [48].

In relation to the second level, the inquiry mode, we employed a questionnaire with predominantly standardized elements and thus it is certainly comparable to classic Likert scales. Such scales capture the degree of agreement or disagreement with given statements according to various response categories. These response categories always comprise an ordinal form of measurement and thus obtaining interval-level information is not possible. Consequently, and in line with a strict interpretation, only statistical procedures that are designed to process ordinal data can be used. However, irrespective of the nature of the data collected by means of a scale close to the Likert model, another implementation of scale requirements predominates in the methodological social sciences literature: often “scales are used which are only believed to be interval-scaled” [49].

## Conclusions

The public pharmacy market in Germany has been facing increasing deregulation. It was initially expected that this would lead to competitive pressure and to an increased competitive orientation on the part of public pharmacies. However, this study indicates that the healthcare reforms of the last 10 years have not necessarily and not generally increased the orientation of public pharmacists towards competition and the application of strategic management. Instead, whilst we find public pharmacies which are rather active and customer-orientated, others behave and feel as if they still live in the era of monopolies in their local pharmaceutical markets. Customer-orientated pharmacies try to bind their customers to them (customer loyalty), show a higher level of a proactive attitude and future orientation, and invest in efforts to increase customer satisfaction. This study confirms that these pharmacies have a higher turnover and a (slightly) higher profit margin. As our analysis predominantly shows correlations but no causality, we must be careful not to over-interpret these findings. However, it seems quite reasonable to conclude that public pharmacies can increase their turnover and their profit margins by showing a more strategic and management-oriented attitude. Thus, pharmacists could benefit from training programmes in entrepreneurship and management.

On the basis of this study, we also recognize that there is no North–South or East–West dichotomy with any outstanding influence. It seems that the “global” location of the pharmacy does not strongly determine economic success. Only the success factor “Sales Profitability” depends on the location, i.e. “West Pharmacies” (N = 218) declare a higher sales profitability than “East Pharmacies” (N = 52). Based on these findings, we can conclude that there is scope for successful business in public pharmacies in every state in Germany. Whether a public pharmacy is profitable seems to depend more on the business-orientation of its owner than on its location in the North/South or West/East.

Finally, we can conclude that even under increased pressure from future healthcare reforms, there is still sufficient space to serve the catchment population and make a profit if public pharmacists learn the essentials of strategic management.

### Ethical clearance

According to German standards, there is no need for ethical clearance for this research as data from anonymous questionnaires are not subject to any ethical approval (Bundesdatenschutzgesetz, BDSG §3, Abs 1 + Abs 6 and §4 Abs. 3). We contacted pharmacy owners with a letter and asked for their views and insights. They could answer or not. No direct observation or impact on human beings was involved.

## Competing interests

The authors have not received reimbursements, fees, funding, or salaries from any organization that may in any way gain or lose financially from the publication of this manuscript, nor will they in the future. The research was not financed by any organisation mentioned in this manuscript. We do not hold any stocks or shares in an organization that may in any way gain or lose financially from the publication of this manuscript, neither now nor in the future. In addition we do not perceive any financial or non-financial competing interests in relation to this manuscript?

## Authors’ contributions

JH carried out the empirical study, prepared the analysis and drafted the first version of the paper. SF contributed to the study design, the mathematical analysis and the final writing of the paper. Both authors read and approved the final manuscript.

## Pre-publication history

The pre-publication history for this paper can be accessed here:

http://www.biomedcentral.com/1472-6963/13/407/prepub

## Supplementary Material

Additional file 1 Questionnaire. Click here for file
