# Supplementary material for: Competition in the German pharmacy market: an empirical analysis
Source: BMC Health Serv Res. 2013 Oct 10;13:407. doi: 10.1186/1472-6963-13-407 (PMC3856528; doi:10.1186/1472-6963-13-407)
Supplement: Additional file 1 — Questionnaire. [file 1472-6963-13-407-S1.doc]

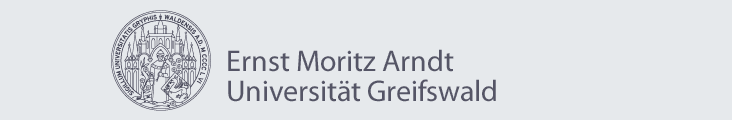


Dipl.-Kfm. Jörg G. Heinsohn Univ.-Professor Dr. rer. pol. Steffen Fleßa

Am Sudedeich 3 Lehrstuhl für Allgemeine Betriebswirtschaftslehre

19273 Soltow und Gesundheitsmanagement

Friedrich-Loeffler-Str. 70

17489 Greifswald

**Questionnaire**

# Competition and health care reform of the pharmaceutical market

**Questionnaire**

# Competition and health care reform of the pharmaceutical market

The questionnaire starts with some questions concerning your competitive situation and your experiences. Please take some time to answer the questions as accurately and extensively as possible. If some answers only can be estimated, we would nevertheless appreciate to get your appraisement.

1. **Since when have you been a self-employed pharmacist?***Please tick the correct answer.* Less than 1 year
   Between 1 and 5 years
   Between 5 and 10 years
   More than 10 years

**1**

**2**

**3**

**4**

1. **Since when does your pharmacy exist at the current site?***Please tick the correct answer.* Less than 1 year
   Between 1 and 5 years
   Between 5 and 10 years
   More than 10 years

**1**

**2**

**3**

**4**

1. **Did you establish your pharmacy by yourself or did you take it over?***Please tick the correct answer.* Own establishment
   Continuation of family property (heritage, handover etc.)
   Takeover of a pharmacy owned by others
   Other

**1**

**2**

**3**

**4**

1. **Do you run several pharmacies, respectively subsidiaries?***Please tick the correct answer.* No, just one …......
   Two, since________
   Three, since________
   Four, since________

**1**

**2**

**3**

**4**

1. **In which federal state is/are your pharmacy/ies located?***Please tick the correct answer.* Bavaria Baden–Wurttemberg Berlin
   Brandenburg Bremen Hamburg
   Hesse Mecklenburg-West Pomerania Lower Saxony
   North Rhine-Westphalia Rhineland-Palatinate Saarland
   Saxony Saxony-Anhalt Schleswig-Holstein
   Thuringia

**7**

**8**

**9**

**10**

**11**

**12**

**13**

**14**

**15**

**16**

**1**

**2**

**3**

**4**

**5**

**6**

1. **How many inhabitants does your current site approximately have?***Please tick the correct answer.* Less than 5.000 inhabitants
   Between 5.000 and 10.000 inhabitants
   Between 10.000 and 50.000 inhabitants
   Between 50.000 and 100.000 inhabitants
   Between 100.000 and 500.000 inhabitants
   More than 500.000 inhabitants
2. **a) Did you change the location during the time of your self-employment, respectively consider it?**
   *Please tick the correct answer.* Yes, once
   Yes, several times
   No
   **b) If you have answered with „yes“, please name the reasons for your decision/considerations to change the location:**

**1**

**2**

**3**

**4**

**5**

**6**

**1**

**2**

**3**

**1**

**2**

**3**

**4**

**5**

**6**

**1**

**2**

**3**

**4**

**5**

1. **With how many pharmacies do you compete with at your site?**

*Please tick the correct answer.* No competitor …………………………………………………………………..............
1 competitor.....................................................................................................................
2 to 3 competitors………………………………………………………………………
4 to 6 competitors……………………………………………………………………….
7 to 10 competitors………………………………………………………………………
More than 10 competitors……………………………………………………………….

1. **How do you consider your competitive situation on the pharmacy market in general? Would you describe it as predominantly…**
   *Please tick the correct answer.*
   Very good
   Good
   Satisfying
   Difficult
   Very difficult
2. **How high is the competitive pressure on your pharmacy which is caused by your main competitors?**
   *Please tick the correct answer.*
   Very low
   Low
   Average
   High
   Very high
3. **Since which year competitive pressure (see question 10) has been changing strongly?**

**1**

**2**

**3**

**4**

**5**

Since year _____

1. **Please evaluate the competitive situation of your pharmacy compared to your main competitors. Would you describe the competitive situation on the field of… as…**
   *Please tick the correct answer in each line.*

Clearly weaker Weaker Equal Superior Clearly superior

Innovative capacity
Product quality
Customer advisory service
Customer service (After-Sales)
Benefits in costs
Financial resources
Profit situation
Marketing/awareness level

**1**

**2**

**3**

**4**

**5**

**1**

**2**

**3**

**4**

**5**

**1**

**2**

**3**

**4**

**5**

**1**

**2**

**3**

**4**

**5**

**1**

**2**

**3**

**4**

**5**

**1**

**2**

**3**

**4**

**5**

**1**

**2**

**3**

**4**

**5**

**1**

**2**

**3**

**4**

**5**

1. **How do you consider your conduct towards your main competitors? Would you consider yourself as predominantly…**
   *Please tick the correct answer.*
   Very defensive
   Defensive
   Neutral
   Offensive
   Very offensive

**1**

**2**

**3**

**4**

**5**

1. **How do you consider your competitive situation compared to mail order selling and business models which are offered e.g. by chemists?**

*Please tick the correct answer in each line.*

Very good Good Satisfactory Difficult Very difficult

Mail order selling

**1**

**2**

**3**

**4**

**5**

**1**

**2**

**3**

**4**

**5**

**1**

**2**

**3**

**4**

**5**

Chemists

Franchise (e.g. Doc Morris)

1. **a) Could you imagine, respectively are you planning, to cooperate with other pharmacists or pharmaceutical companies in future to gain competitive advantages?**
   *Please tick the correct answer.*
   Generally not
   Probably not
   Maybe selectively
   In principle yes
   Already practised experience
   **b) If you have already gained experience with cooperations, please describe them:**

**1**

**2**

**3**

**4**

**5**

1. **How did the net sales of your company change on average**  **in the years 2004 – 2007?**
   *Please tick the correct answer.*
   Decreased strongly (>-10%)
   Decreased (-10% to -1%)
   Unchanged
   Increased (1% to 5%)
   Increased strongly (5% to 10%)
   Increased very strongly (>10%)

**1**

**2**

**3**

**4**

**5**

**6**

1. **Which average profit-turnover ratio ( ^Profit^ x 100) (before tax and imputed entrepreneurial profit) did your company achieve in the last three years?**
   *Please tick the correct answer. If there is no exact data available, please estimate.*
   Negative (-10% to -1%)……………………………………………………………….
   Break-even…………………………………………………………………………….
   Positive (1% to 5%)……………………………………………………………………
   Strongly positive (5% to 10%)…………………………………………………………
   Very strongly positive (>10%)…………………………………………………………

**1**

**2**

**3**

**4**

**5**

**Turnover**

1. **Which size did your company reach?**
   *Please tick the category of net sales by the end of the last trading year 2007 and enter the shares (in percent) of the following sales segments of turnover in total.*

Turnover of end-consumers concerning non-prescription drugs

**OTC**

(in %)

Turnover from prescription of *non-prescription* drugs

**OTX**

(in %)

Turnover in total

Category of turnover

(in thousand euros)

< 1250 □

> 1250 <= 2400 □

< 2400 □

Turnover from

complementary range (toiletries*/*dietary supplement)

**Self-service products** (in %)

Turnover from prescription of *ethical* drugs

(in %)

1. **How would you characterize the average spending power of your clients?**
   *Please tick the correct answer.*
   Low
   Low to medium
   Medium
   Medium to high
   High
2. **How often do you recommend over-the-counter drugs for self-medication during customer advisory service?**
   *Please tick the correct answer.*
   Never
   Rarely
   Occasionally
   Often
   Very often

**1**

**2**

**3**

**4**

**5**

**1**

**2**

**3**

**4**

**5**

1. **Please name the top-selling sector for self-medication of your pharmacy:***Please tick the correct answer.* Cardio/circulatory complaints Infection/disease of immune system Dermatics
   Gastrointestinal complaints Tranquilliser and moodlifter Immunostimulation
   Vein problems/haemorrhoids Kidney/bladder/genito-urinary system Antiallergics
   Eye/ear/mouth Strengthening/prevention/vitalization Other
   Analgesics/Antirheumatic agents Vitamins/minerals/micronutrients

**1**

**2**

**3**

**4**

**5**

**6**

**7**

**8**

**9**

**10**

**11**

**12**

**13**

**14**

1. **Related to ingredients, how can products for self-medication, recommended by you, be characterized? Do you recommend...***Please tick the correct answer in each line.*

Not at all To a small extent Occasionally Often Very often

Chemically defined active ingredients
Herbal active ingredients
Naturopathic products
Homeopathics
Dietary supplements
Other

**1**

**2**

**3**

**4**

**5**

**1**

**2**

**3**

**4**

**5**

**1**

**2**

**3**

**4**

**5**

**1**

**2**

**3**

**4**

**5**

**1**

**2**

**3**

**4**

**5**

**1**

**2**

**3**

**4**

**5**

1. **To what extent do you use measures of differentiation to realize competitive advantages within the range of self-medication? For this purpose I use...**
   *Please tick the correct answer in each line.*

Not at all To a small extent Occasionally Often Very often

Therapeutic quality ….. …. … … …………...
Product quality ……. … … … …………...
Pharmaceutical forms …. …. … … ...
Convenience of/information about handling … … … ...
Packaging design/free supplements ….. …. … … ...
Brand loyalty …. …. … … ..
Individual customer advisory service…. … … … ..
Product-related services …. …. … … ..
Customer service (including after sales) … … … ...
Innovative marketing …. … … … ..

**1**

**2**

**3**

**4**

**5**

**1**

**2**

**3**

**4**

**5**

**1**

**2**

**3**

**4**

**5**

**1**

**2**

**3**

**4**

**5**

**1**

**2**

**3**

**4**

**5**

**1**

**2**

**3**

**4**

**5**

**1**

**2**

**3**

**4**

**5**

**1**

**2**

**3**

**4**

**5**

**1**

**2**

**3**

**4**

**5**

**1**

**2**

**3**

**4**

**5**

Online trading …. …. … … ...
Other measures …. …………………………………...
*Please name them*: ...................................................................................................

**1**

**2**

**3**

**4**

**5**

1. **Are you planning to have your pharmacy certified according to DIN-EN-ISO 9002?**
   *Please tick the correct answer.*
   Generally not
   Probably not
   Probably yes
   Certainly yes
   Already done

**1**

**2**

**3**

**4**

**5**

**1**

**2**

**3**

**4**

**5**

1. **To what extent do you use the following ways of communication concerning products for self-medication? For this purpose I use...**
   *Please tick the correct answer in each line.*

Not at all To a small extent Occasionally Often Very often

Traditional advertising (advertisements, and the like) … … ……...
Sales promotions at point of sale … … … …….......
Scientific information … … … …….......
Scientific information … … … …….......
Information magazines (e.g. Apothekenumschau) … … ……...
Public relations/promotion weeks … … … ……......
Sponsorship/charitable campaigns … … … ……......
Seminars/training events … … … ……......
Exhibitions/fairs … … … ……......
Leaflets/handouts … … … ……......
Samples and giveaways … … . … ……......
Own products/private label … … … ……......
Other measures … … … ……...
*Please name them*: ...............................................................................................

**1**

**2**

**3**

**4**

**5**

**1**

**2**

**3**

**4**

**5**

**1**

**2**

**3**

**4**

**5**

**1**

**2**

**3**

**4**

**5**

**1**

**2**

**3**

**4**

**5**

**1**

**2**

**3**

**4**

**5**

**1**

**2**

**3**

**4**

**5**

**1**

**2**

**3**

**4**

**5**

**1**

**2**

**3**

**4**

**5**

**1**

**2**

**3**

**4**

**5**

**1**

**2**

**3**

**4**

**5**

**1**

**2**

**3**

**4**

**5**

1. **a) Are you planning to use or already testing innovative distribution channels, like internet mail order selling?**
   *Please tick the correct answer.*
   Generally not
   Probably not
   Maybe selectively
   In principle yes
   Already practised successfully
   **b) Please describe your experiences or considerations:**

**1**

**2**

**3**

**4**

**5**

1. **a) Are you planning to extend the customer focus of your pharmacy by means of e.g. personnel training?**
   *Please tick the correct answer.*
   Generally not
   Probably not
   Probably yes
   Certainly yes
   Already practised successfully
   **b) Are you also planning some events for patients concerning health related topics?**Generally not
   Probably not
   Probably yes
   Certainly yes
   Already practised successfully  **c) Which measures for the improvement of customer focus of your staff would you constitute as especially promising?**

**1**

**2**

**3**

**4**

**5**

**1**

**2**

**3**

**4**

**5**

1. **a) The pharmacy market is already subject to a large number of regulations. How would you rate the new regulations in recent years for your business development?**
   *Please tick the correct answer.*
   Very obstructive
   Rather obstructive
   Undecided
   Rather conducive
   Very conducive

**1**

**2**

**3**

**4**

**5**

**1**

**2**

**3**

**4**

**5**

**1**

**2**

**3**

**4**

**5**

**b) Please name the settlements you consider as particularly positive or negative:**

1. **According to the intention of the legislator, the health care reform shall solve many problems of the health care system. What is your personal opinion about this? Health care reform will ... the situation of your pharmacy:**
   *Please tick the correct answer.*
   Strongly improve
   Improve
   Not change
   Rather worsen
   Strongly worsen

**1**

**2**

**3**

**4**

**5**

1. **How do you evaluate the prohibition of minority interests (including joint-stock companies) on the pharmacy market?***Please tick the correct answer.*
   Very good (should definitely be maintained)
   Good
   Undecided
   Rather negative
   Very negative (should definitely be changed)

**1**

**2**

**3**

**4**

**5**

*Finally, here are a couple of questions concerning yourself and your company.*

1. **Which sex do you have?**
   Female Male

**1**

**2**

1. **How old are you?**___________years
2. **Which marital status do you have?**
   Single Domestic partnership
   Married Separated
   Widowed Divorced

**1**

**2**

**3**

**4**

**5**

**6**

1. **How many employees work in your pharmacy/branch pharmacy(ies)?**

***(Please convert part-time employees proportionally into full-time employees)***
___________ employees

Subsidiary(ies): First_____Second_____Third____employees

1. **How many family members work in your pharmacy?**
   ___________family members
2. **Are you planning to extend the number of your employees in near future?**
   *Please tick the correct answer.*
   Generally not
   Probably not
   Probably yes
   Certainly yes

**1**

**2**

**3**

**4**

1. **Are you planning cost or staff savings in near future?***Please tick the correct answer.*
   Generally not
   Probably not
   Probably yes
   Certainly yes

**1**

**2**

**3**

**4**
